# Highly active repeat-mediated recombination in the mitogenome of the holoparasitic plant *Aeginetia indica*

**DOI:** 10.3389/fpls.2022.988368

**Published:** 2022-09-21

**Authors:** Yan Zhong, Runxian Yu, Jingfang Chen, Ying Liu, Renchao Zhou

**Affiliations:** ^1^ State Key Laboratory of Biocontrol and Guangdong Provincial Key Laboratory of Plant Resources, School of Life Sciences, Sun Yat-sen University, Guangzhou, China; ^2^ State Key Laboratory of Systematic and Evolutionary Botany, Institute of Botany, The Chinese Academy of Sciences, Beijing, China; ^3^ College of Life Sciences, University of Chinese Academy of Sciences, Beijing, China

**Keywords:** *Aeginetia indica*, mitochondrial genome, repeat-mediated recombination, intraspecific variation, HGT

## Abstract

Mitogenomes of most flowering plants evolve slowly in sequence, but rapidly in structure. The rearrangements in structure are mainly caused by repeat-mediated recombination. However, patterns of repeat-mediated recombination vary substantially among plants, and to provide a comprehensive picture, characterization of repeat-mediated recombination should extend to more plant species, including parasitic plants with a distinct heterotrophic lifestyle. Here we assembled the mitogenome of the holoparasitic plant *Aeginetia indica* (Orobanchaceae) using Illumina sequencing reads. The mitogenome was assembled into a circular chromosome of 420,362 bp, 18,734 bp longer than that of another individual of *A. indica* which was assembled before as a linear molecule. Synteny analysis between the two mitogenomes revealed numerous rearrangements, unique regions of each individual and 0.2% sequence divergence in their syntenic regions. The *A. indica* mitogenome contains a gene content typical of flowering plants (33 protein-coding, 3 rRNA, and 17 tRNA genes). Repetitive sequences >30 bp in size totals 57,060 bp, representing 13.6% of the mitogenome. We examined recombination mediated by repeats >100 bp in size and found highly active recombination for all the repeats, including a very large repeat of ~16 kb. Recombination between these repeats can form much smaller subgenomic circular chromosomes, which may lead to rapid replication of mitochondrial DNA and thus be advantageous for *A. indica* with a parasitic lifestyle. In addition, unlike some other parasitic plants, *A. indica* shows no evidence for horizontal gene transfer of protein-coding genes in its mitogenome.

## 1 Introduction

The mitogenomes of most flowering plants evolve very slowly in sequence while very rapidly in genome rearrangement (Palmer and Herbon, 1988; Drouin et al., 2008; Gualberto and Newton, 2017; Sloan et al., 2017). The frequent rearrangement in the mitogenomes is thought to stem from recombination between repeated sequences, which are common in the mitogenomes of flowering plants (Alverson et al., 2011; Cole et al., 2018; Kozik et al., 2019). For a circular chromosome, isomeric forms of the circle would form when a repeat pair is present in reverse orientation; alternatively, two small circular chromosomes (subgenomes) would be expected when a repeat pair is present in direct orientation (Kubo and Newton, 2008). As expected, the presence of both direct and reverse repeat pairs would greatly enrich the mitochondrial DNA conformations. Repeats of intermediate length (100-1,000 bp) are thought to be subject to not-infrequent recombination (Maréchal and Brisson, 2010; Woloszynska, 2010; Kozik et al., 2019), while large repeats (>1,000 bp) are expected to generate equimolar or nearly equimolar recombined molecules in some plant species (Maréchal and Brisson, 2010), such as *Ginkgo* (Guo et al., 2016) and monkeyflower (Mower et al., 2012a). However, this is not the case for *Nymphaea colorata*, which have some large repeats but with very low recombination frequency (Dong et al., 2018). At the same time, moderate to considerable recombination frequencies were also found for small repeats (< 100 bp) in some plant species, for example 14.7% for a 55-bp repeat pair of *Picea abies* (Sullivan et al., 2020) and 24.1% for a 75-bp repeat pair of *Viscum scurruloideum* (Skippington et al., 2015). In the mitogenome of *V. scurruloideum*, the length of short repeats (30-100 bp) was positively correlated with recombination rate (Skippington et al., 2015). In contrast, little or no evidence for ongoing recombination mediated by repeats ranging from 100 to 800 bp in the mitogenomes of two *Monsonia* species (Cole et al., 2018).

Recombination mediated by small or medium repeats is usually of low frequency and symmetrical stoichiometry between different conformations is rare (Arrieta-Montiel et al., 2009). Being less well studied, the biological significance of this stoichiometric variation in different conformations is unclear. The degree of stoichiometry equality may potentially affect the rate of DNA replication, gene order, gene expression patterns, and further environmental adaptability. So far, the relationships between repeats and repeat-mediated recombination in the mitogenomes of plants remain elusive. Therefore, mitogenome sequencing from more plants are needed to generalize patterns of their mitogenome evolution, including the recombination activity of repeats.

Parasitic plants consist of ~1% flowering plants and have independently evolved at least 12 or 13 times (Westwood et al., 2010; Nickrent, 2020). They partially or completely obtain water and nutrients from their hosts *via* haustoria due to partial or complete loss of the capability of photosynthesis (Petersen et al., 2020). Plastid genomes of parasitic plants usually exhibit reduced size and gene content, and increased AT content and evolutionary rate (Wicke and Naumann, 2018). Parasitic plants also show deeply altered nuclear genome architecture, such as genome size expansion (>100 Gb in *Viscum*) (Marie and Brown, 1993), substantial gene loss (*Cuscuta* and *Sapria*) (Sun et al., 2018; Cai et al., 2021) and frequent horizontal gene transfer (HGT) from their hosts (Kado and Innan, 2018). Although rarely characterized, mitogenomes of some parasitic plants also show unusual features, such as minicircular chromosomes (Yu et al., 2022), extreme size reduction, gene loss and high substitution rate (Skippington et al., 2015); extreme heteroplasmy (Yu et al., 2022), and rampant HGT (Petersen et al., 2020). However, these unusual features are not universal to parasitic plants, for example, the hemi-parasitic plant *Castilleja paramensis* appears to have a mitogenome typical of flowering plants (Fan et al., 2016), and mitochondrial substitution rates of parasitic plants are not always higher than those of non-parasitic plants (Zervas et al., 2019).

*Aeginetia* is a holoparasitic genus of Orobanchaceae, mostly distributed in tropical Asia (Kuijt, 1969; POWO, 2022). This genus comprises four species, of which *A. indica* is the most widely distributed species. The hosts of *A. indica* are usually plants from Poaceae like *Miscanthus* and *Saccharum* (sugar-cane) (Kuijt, 1969). Previous studies showed that *A. indica* had lost almost all photosynthesis-related genes in its plastome (Chen et al., 2020) and 84 nuclear genes of *A. indica* had been obtained *via* horizontal gene transfer (HGT) from its hosts (Kado and Innan, 2018). A recent study attempted to assemble the mitogenome of *A. indica*, but failed to get a complete assembly (Choi and Park, 2021). Thus, its mitogenome structure remains unknown.

In this study, we successfully assembled a complete circular-mapping mitogenome of *A. indica* using Illumina sequencing reads. We found highly active repeat-mediated recombination in its mitogenome, implying numerous alternative genomic and subgenomic conformations in this holoparasitic plant, which may be advantageous for rapid replication of its mitochondrial DNA. In addition, unlike some other parasitic plants, *A. indica* shows no evidence for HGT of protein-coding genes in its mitogenome.

## 2 Results and discussion

### 2.1 The mitogenome of *A. indica* contains a gene content typical of flowering plants

**Figure 1:**
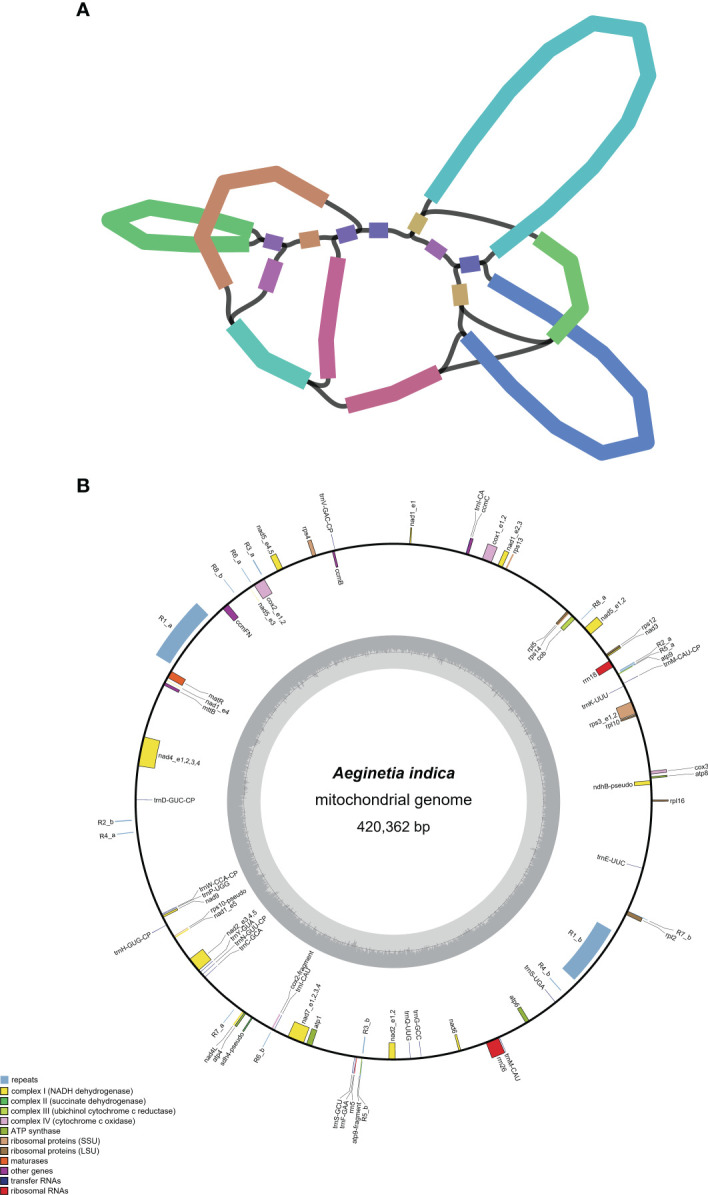
The mitogenome of *Aeginetia indica*. **(A)**, A network of the mitochondrial contigs of *Aeginetia indica* visualized in Bandage. **(B)**, Gene map. Genes shown outside the outer circle are transcribed counterclockwise, whereas those inside the outer circle are transcribed clockwise. The darker and light shading inside the inner circle indicates GC and AT content, respectively. The eight largest repeats (R1-R8) are also shown in blue in this figure.

Genome assembly using Illumina reads generated 614,229 contigs > 127 bp in length for *A. indica*. We obtained 17 putative mitochondrial contigs by filtering out the contigs with very high (putative plastid contigs) and low (putative nuclear contigs) sequening depth. When visualized in Bandage, these putative mitochondrial contigs are connected to a network, and several contigs with four links to other contigs likely represent repeats in the *A. indica* mitogenome (**Figure 1A**). Many different conformations of the *A. indica* mitogenome can be inferred from the contig network. For the illustrative purpose, we selected for display a full-genome conformation consisting of all these contigs (**Figure 1B**). The mitogenome size is 420,362 bp, and its overall GC content of is 43.5%, similar to that found in most other angiosperms (Mower et al., 2012b; Skippington et al., 2015). The sequencing depth across the mitogenome is relatively even (**Figure S1**), with an average depth of 357 ×, validating the accuracy of our assembly.

The *A. indica* mitogenome contains 33 protein-coding, 3 rRNA, and 17 tRNA genes (**Table S1**). The 33 protein-coding genes includes 24 core genes usually present in seed plants (Adams et al., 2002) and nine genes variably present in seed plants. The protein-coding genes contain 16 *cis*-spliced and six *trans*-spliced introns. As shown in **Table S2**, the mitogenomes of all eight Orobanchaceae species (including the autotrophic plant *Lindenbergia philippensis*) have highly similar gene contents, with occasional loss and/or psedogenization of several genes variably present in seed plants. Compared with its close relative *Castilleja paramensis*, *A. indica* has lost one gene *sdh3*. In addition, *rps10* is intact in *C. paramensis* while pseudogenized in *A. indica*, and *rpl2* is intact in *A. indica* while pseudogenized in *C. paramensis*. In addition to the three introns (*cox2*-i1, *nad7*-i3, and *rpl2*-i1), which were also missing in seven other Orobanchaceae species (Fan et al., 2016), one more intron (*rps10*-i1) was lost in *A. indica* than *Castilleja paramensis* due to pseudogenization of *rps10*.

Available complete plant mitogenomes suggests no evolutionary correlation between the type of parasitic lifestyle and mitogenome size and structure (Petersen et al., 2020). Also, the effects of the parasitic lifestyle on mitogenome gene content of plants remain unclear, because there are so few well-annotated mitogenomes of parasitic plants. Although the mitochondrial genomes of the mistletoes *Viscum* (Petersen et al., 2015; Skippington et al., 2015) exhibit substantial gene loss, other parasitic plants do not show significant gene loss, including seven Orobanchaceae species (Fan et al., 2016), *Cynomorium coccineum* (Bellot et al., 2016), *Rafflesia lagascae* (Molina et al., 2014), *Tolypanthus maclurei* (Yu et al., 2021), *Cuscuta* (Lin et al., 2022), *Rhopalocnemis phalloides* (Yu et al., 2022), and *A. indica* in this study. This suggests mitochondrial gene loss may not be directly associated with the parasitic lifestyle in most parasitic plants.

### 2.2 Intraspecific variation in the mitogenomes of *A. indica*

**Figure 2:**
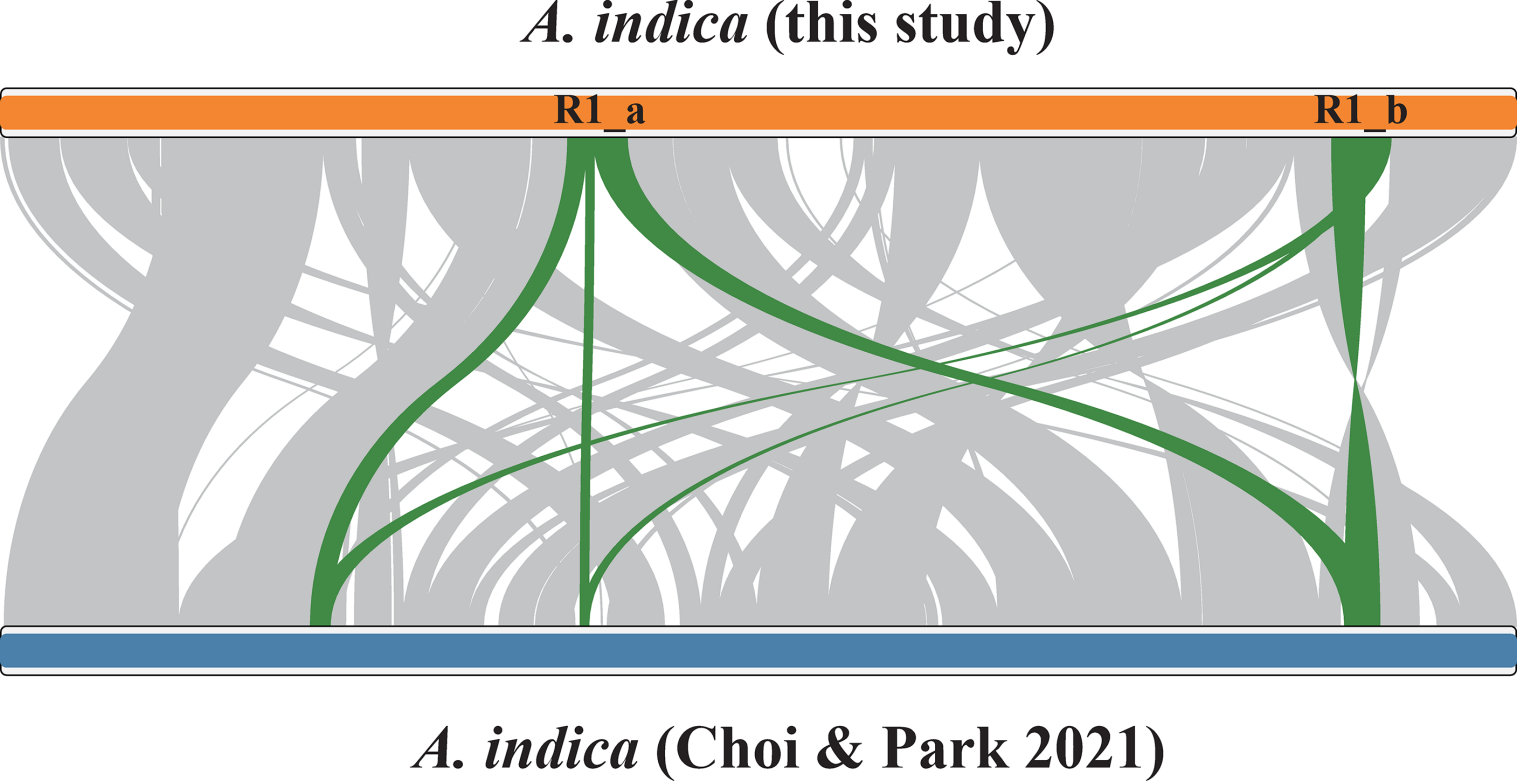
Numerous rearrangements between the two *Aeginetia indica* mitogenomes. The green curve corresponds to the ~16 kb repeat pair (R1) in the *A. indica* mitogenome of this study.

Compared with the mitogenome of another individual of *A. indica*, which was collected from Jeju Island, Korea and comprised a linear molecule with a total length of 401,628 bp (Choi and Park, 2021), the mitogenome in this study is 18,734 bp larger in size. The syntenic analysis of the two *A. indica* mitogenomes revealed numerous rearrangements (**Figure 2**). 389,843 bp out of 420,362 bp (92.7% of the mitogenome in this study) can be aligned and the alignable regions had an identity greater than 99%. The alignable regions between the two individuals contains 864 nucleotide substitutions (0.2% divergence). The unalignable sequences are all in the intergenic regions. There are 33 and 25 unalignable regions with a total length of 49,642 bp and 30,512 bp, respectively, in the mitogenomes of the two individuals. The largest repeat, R1, (see section 2.3 below) in the mitogenome of this study is divided into three parts in the mitogenome of the other individual (**Figure 2**).

The *A. indica* mitogenome reported by Choi and Park (2021) annotated two more protein-coding genes (*rps10* and *atpI*) than that in this study. *rps10* was annotated as a pseudogene in this study due to substantial truncation, and careful sequence inspection for the mitogenome sequence reported by Choi and Park (2021) found an opening reading frame (ORF) of only 165 bp for this gene, much shorter than ORF length of this gene in other angiosperms (~360 bp). So it must be a pseudogene as well. *atpI* was suggested to be acquired by horizontal gene transfer (Choi and Park, 2021), but in fact it is of plastid origin and an only 216 bp gene fragment (~750 bp in other angiosperms) was found in both this study and Choi and Park (2021). Therefore, the mitochondrial protein-coding gene content is actually the same for the two individuals of *A. indica*.

We annotated 17 tRNAs and three rRNAs in the *A. indica* mitogenome (**Table S1**), two more tRNAs than those annotated in Choi and Park (2021). We reannotated the tRNAs in the *A. indica* mitogeome reported by Choi and Park (2021) using the same method. We found the mitogenome reported by Choi and Park (2021) had the same 17 tRNAs, plus one more private tRNA (*trnS-GGA*) in an unalignable region.

Previous studies, albeit very few, have revealed the existence of intraspecific variation in plant mitogenomes, including structural rearrangements in *Beta vulgaris* (Darracq et al., 2011), copy number variation in *Arabidopsis thaliana* (Wu et al., 2020), chromosome presence/absence in *Silene noctiflora* (Wu et al., 2015; Wu and Sloan, 2019), and nucleotide variations in *Oryza rufipogon* and *O. sativa* (He et al., 2020). The numerous rearrangements and abundant sequence variations between the mitogenomes of Choi and Park (2021) and this study suggests that there is a considerable intraspecific mitogenomic variation in *A. indica*.

### 2.3 Highly active repeat-mediated recombination

**Figure 3:**
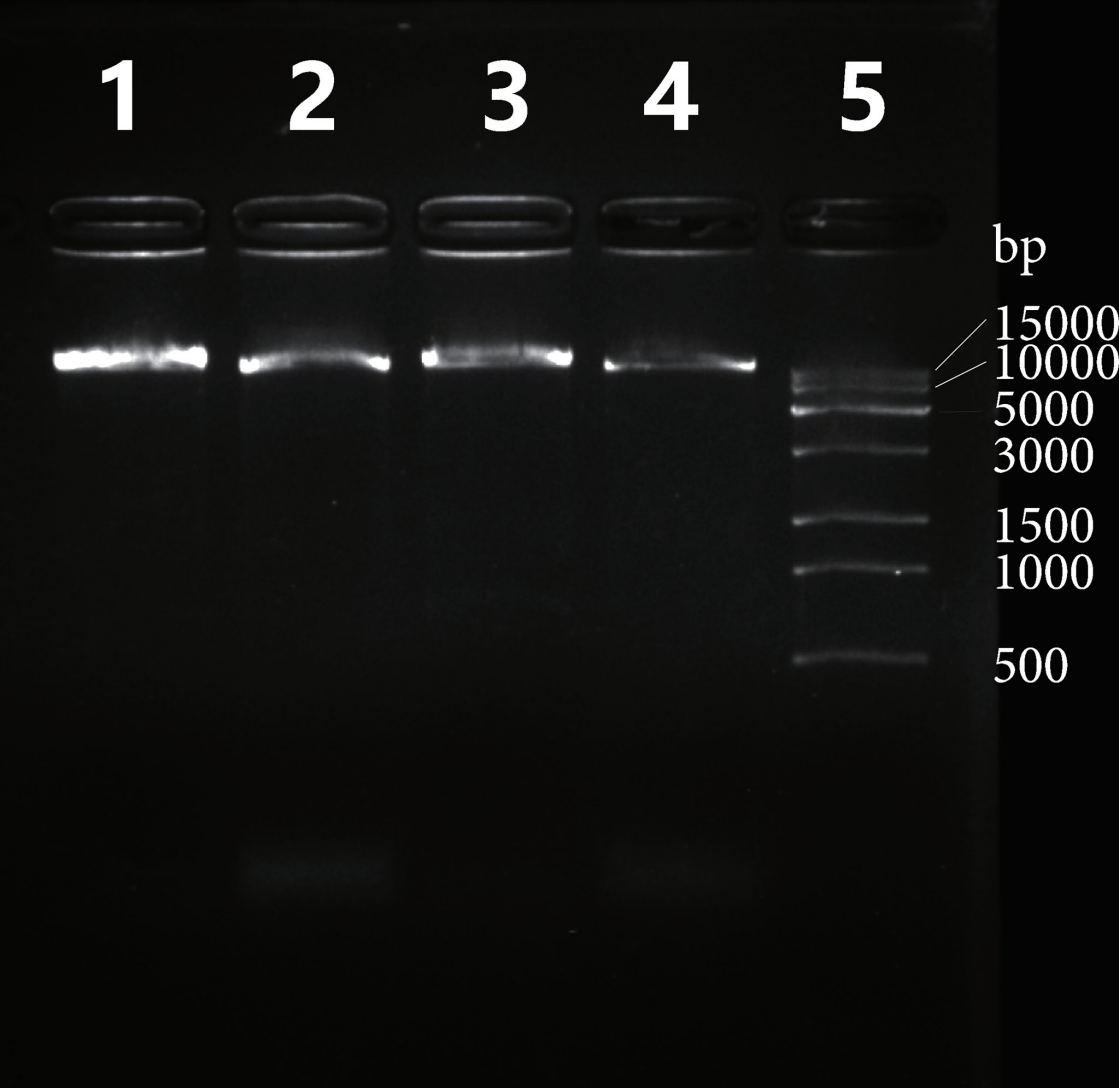
PCR evaluation of recombination mediated by a ~16 kp repeat pair (R1) in the *Aeginetia indica* mitogenome. Shown are results of DNA gel electrophoresis of amplified PCR products. Lane 1: PCR amplification of R1_a and flanking regions (primer: a_F and a_R); Lane 2: PCR amplification of R1_b and flanking regions (primer: b_F and b_R); Lane 3: PCR amplification for the recombined conformation 1 (primer: a_F and b_F); Lane 4: PCR amplification for the recombined conformation 2 (primer: a_R and b_R); Lane 5: DNA ladder. Expected sizes for these PCR products are 17734, 17589, 17809 and 17514 bp for Lane 1, 2, 3 and 4, respectively.

The *A. indica* mitogenome contains 377 repeat units ≥ 30 bp in size, which form 846 repeat copies and a total of 57,060 bp repetitive sequences, covering 13.6% of the mitogenome (**Supplementary Excel file 2**). All repeat units >100 bp in size, including one large repeat unit (R1, 16,366 bp) and seven relatively small repeat units (R2-R8, 111-237 bp), have two copies (**Table 1**). These eight repeat pairs are all located in the intergenic regions (**Figure 1B**). We analyzed recombination activity of the eight repeat pairs. The largest repeat pair can mediate recombination, as verified by the results of PCR amplification (**Figure 3**). Recombination mediated by seven other repeat pairs were verified by mapping Illumina reads to the reference and potentially recombined conformations.

**Table 1:** Repeat-mediatied recombination in the *Aeginetia indica* mitogenome.

Repeat pair	Repeat length(bp)	Repeat type	Naa^a^	Nbb^a^	Nab^a^	Nba^a^	Recombination frequency
R2	237	direct	235	206	167	163	42.8%
R3^b^	233	reverse	177		150		45.9%
R4	186	reverse	278	250	272	286	51.4%
R5^b^	181	direct	449		358		44.4%
R6	127	direct	590	600	662	584	51.2%
R7	111	direct	548	459	499	426	47.9%
R8	111	direct	521	447	473	442	48.6%

^a^Naa and Nbb are the number of reads mapped to the reference conformations; Nab and Nba are the number of reads mapped to the recombined conformations. Recombination frequency is calculated as: (Nab+Nba)/(Nab+Nba+Naa+Nbb). ^b^Because R3 and R5 have an overlap of 3 bp for one copy (R3_b and R5_b) and they each have three reference conformations and three recombined conformations, the numbers of reads supporting the reference and recombined conformations for R3 and R5 were summarized here (as shown in **Figure S2**).

Although high-frequency recombinations is often mediated by large repeats in many plant species, this is not necessarily the case in *A. indica*. For all seven small repeat pairs of *A. indica*, we observed very high recombination rates, ranging from 42.8% to 51.4% (**Table 1**), in nearly equimolar amounts (50.0%). Even though recombination rates are not widely characterized in plant species, they are higher than many of the reported fractions of recombination. For example, *Lactuca sativa* and *L. serriola* have a very low fraction of recombinations (~1%-10%) mediated by their short repeats (Kozik et al., 2019), and *Nymphaea colorata* possesses 0.2% and 8.2% recombination frequencies for its two largest repeats (Dong et al., 2018). Evidence of recombination was detected in *Vigna angularis* mitogenome for twelve repeat units of 62-1,215 bp length, with recombination rates ranging from 5% to 33% (Naito et al., 2013). In *Picea abies*, most of the detected repeat pairs showed no to little evidence of repeat-mediated recombination, but a few ranging from 50 bp to 948 bp were found to have highly active recombination, reaching a maximum of ~32% for a 186 bp repeat pair (Sullivan et al., 2020). In addition, four repeat pairs (387-593 bp in length) in the mitogenome of a parasitic mistletoe *Viscum scurruloideum* have 39-58% recombination frequencies, which was considered to be the smallest repeats at recombinational equilibrium in plant mitogenomes (Skippington et al., 2015). The repeats at recombinational equilibrium are even smaller in the mitogenome of *A. indica*. This implies highly active repeat-mediated recombination occurring in the mitogenome of *A. indica*, which is consistent with the numerous rearrangements shown in the collinear analysis (**Figure 2**).

**Figure 4:**
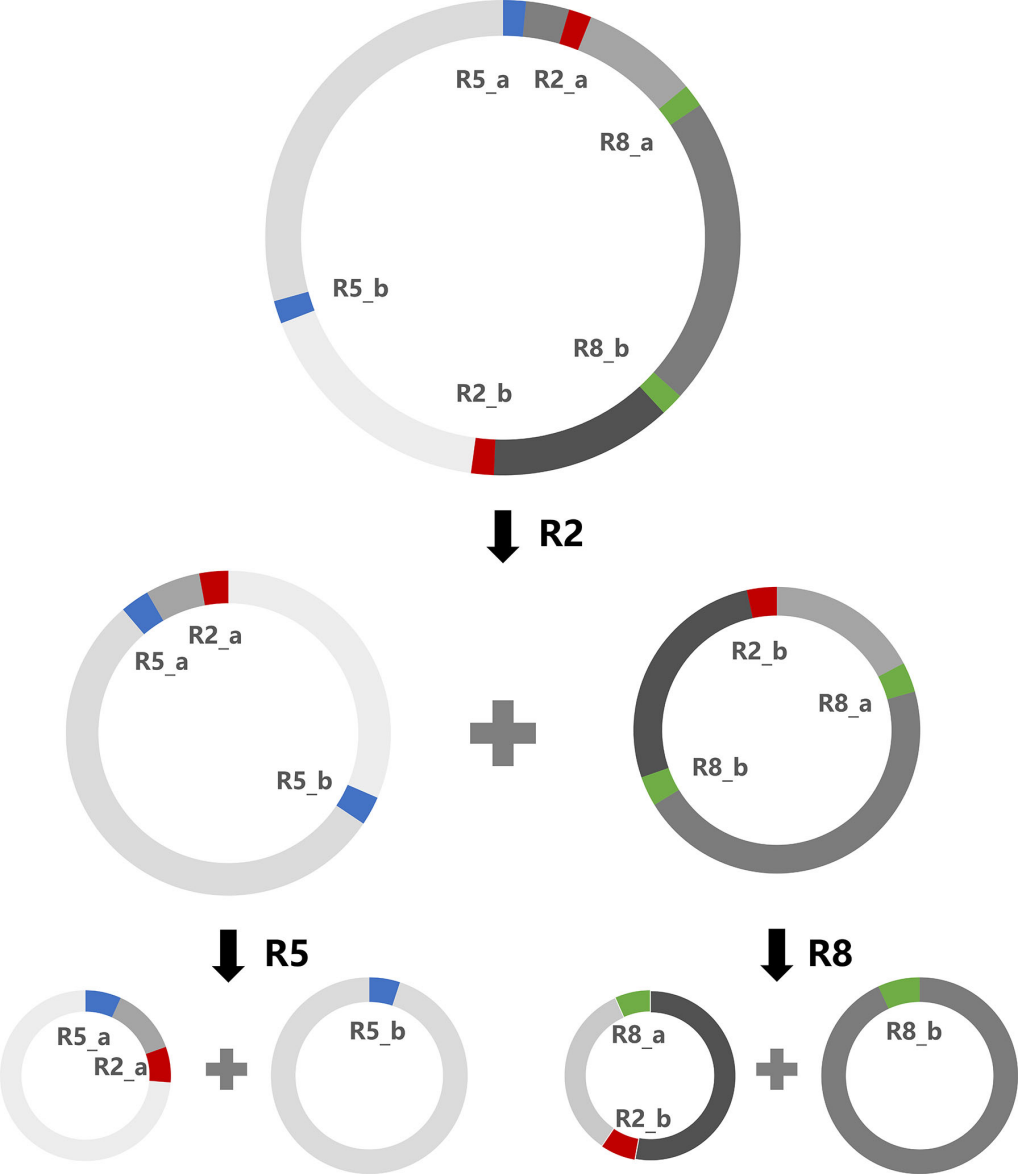
A schematic diagram of multiple alternative mitogenomic conformations formed by repeat-mediated recombination. This diagram shows only one of the possible ways of repeat-mediated recombination. These three repeat pairs (R2, R5 and R8) in the *A. indica* mitogenome are direct repeats.

The *A. indica* mitogenome can form multiple alternative conformations through repeat-mediated recombination. All alternative conformations resulting from repeat-mediated recombination contain identical genomic content. These conformations differ only in the order and orientation of non-repeat regions and repeat regions separating them, and in some cases, also include differences in chromosome number (dividing one big circular chromosome into two or more small circular chromosomes). Here we show one of the possible recombination scenarios (**Figure 4**). The circular chromosome can shift into two small subgenomic circular chromosomes by R2 mediated recombination, and further into four smaller subgenomic circular chromosomes by R5 and R8 mediated recombination. A special case was found for R3 and R5, of which one copy (R3_b and R5_b) has an overlap of 3 bp. Unlike other repeats, R3 and R5 each have three predicted reference conformations and three predicted recombined conformations, as illustrated in **Figure S2**. Through repeat-mediated recombinations, the *A. indica* mitogenome can exist in various conformations, including many small subgenomic circular chromosomes. At the same DNA replication speed, the smaller chromosomes will be expected to replicate faster. Rapid replication of mitochondrial DNA may be beneficial for holoparasitic plants like *A. indica* to cope with a harsh heterotrophic lifestyle.

### 2.4 No to little horizontal and intracellular gene transfer in the *A. indica* mitogenome

Phylogenetic analysis for each of the 33 mitochondrial protein-coding genes revealed that *A. indica* always clustered with other species of Lamiales with high bootstrap support, and that monocots, its potential hosts, formed a distant monophyletic clade (**Supplementary Figure 1**). This is also the case for another parasitic plant from the same family, *Castilleja paramensis*. Therefore, no evidence for HGT was found in the mitogenomes of the two parasitic plants in Orobanchaceae. Some parasitic plants show rampant HGT in their mitogenomes from their hosts (Sanchez-Puerta et al., 2019) and a plausible explanation is that parasitic plants establish intimate contact with their hosts *via* haustoria (Goyet et al., 2019). However, no evidence for HGT in the holoparasitic plant *A. indica*, the hemiparasitic plant *Castilleja paramensis* and two *Cuscuta* species (Anderson et al., 2021) suggests that physical contact between parasitic plants and their hosts is not always associated with host-to-parasitic-plants mitochondrial HGT. On the other hand, the *A. indica* nuclear genome indeed possesses some genes derived from its hosts (Kado and Innan, 2018), which implies that patterns of HGT from hosts to parasitic plants might differ between mitogenomes and nuclear genomes. Why the extent of HGT differ substantially between mitogenomes of parasitic plants, and between the mitogenome and nuclear genome of a parasitic plant deserves further investigation.

In terms of intracellular gene transfer (IGT) between the mitogenome and the plastome of *A. indica*, we found two regions (220 bp and 251 bp) in the mitogenome with hits to the psuedogene ψ*ndhB* in its plastome. BLASTN search of the *A. indica* mitogenome against the plastome of *L. philippensis* revealed a 611-bp hit to the *ndhB* gene of *L. philippensis* (gene length = 2,212 bp). Therefore, we suggest that the pseudogene ψ*ndhB* in the *A. indica* mitogenome was transferred from its plastome and that little IGT has occurred between the mitogenome and the plastome of *A. indica.*

## 3 Materials and methods

### 3.1 Plant sampling, Illumina sequencing and mitogenome assembly

Plant collection and Illumina sequencing of an individual of *Aeginetia indica* were performed as described in (Chen et al., 2020). The individual was collected from Shimentai Forest Park, Yingde, Guangdong, China. 21.4 Gb of 150 bp paired-end reads with an insert size of 300 bp were generated. Raw reads were filtered by Trimmomatic v 0.39 (Bolger et al., 2014) with default parameters. The mitogenome was assembled using Illumina reads in GetOrganelle v1.7 (Jin et al., 2020) with the parameters: -F embplant_mt, -k 57,77,97,117,127, and the mitogenomes of *Liriodendron tulipifera* and *Castilleja paramensis* were used as references. We identified putative mitochondrial contigs when they have a depth of coverage > 100× (based on the depths of several contigs containing the mitochondrial genes), BLASTN (evalue set to 1e-5) hits to the plant mitochondrial databases, or with direct or indirect links (based on the GFA output) to the contigs with mitochondrial hits. By visualizing these potential mitochondrial contigs in Bandage v0.8.1 (Wick et al., 2015), the complete mitogenome could be manually connected into a circular chromosome, but with many alternative conformations of the same genome size. We selected for display one full-genome conformation for subsequent analysis. All Illumina reads were mapped back to the mitogenome using BWA-mem (Li, 2013) with default parameters except -T set to 100 and the depth of coverage across the chromosome was calculated using Samtools v1.9 (Li et al., 2009). To avoid the influence of plastid-derived reads on the sequencing depth of the mitogenome, the plastid genome of *A. indica* (GenBank accession number MN529629) was also used as reference during mapping of Illumina reads. To avoid the influence of nuclear-derived reads, the mapped alignment shorter than 100 bp were filtered.

### 3.2 Mitogenome annotation

The mitochondrial protein-coding genes and rRNAs of *A. indica* were annotated with Geseq (Tillich et al., 2017) and BLASTN with default settings (Camacho et al., 2009). Available mitogenomes of Lamiales species were used as references and manual adjustment was conducted when necessary. Genes that contain one or more premature stop codons or frameshift mutations were considered as pseudogenes. Transfer RNAs (tRNAs) were identified using tRNAscan-SE v2.0.7 (Lowe and Chan, 2016) with the organelle option. Mitochondrial gene maps (including eight repeats >100 bp in size) were plotted by OGDRAW (Greiner et al., 2019).

### 3.3 Collinear analysis with another individual of *A. indica* and comparative analysis with other species in Orobanchaceae

The mitogenome of another individual of *A. indica* characterized by Choi and Park (2021) were used for comparison. This individual was collected from Jeju Island, Korea, which is more than 1,700 km away from the collection site of the individual we studied here. Collinear analysis between the two mitogenomes were conducted in Mummer (Kurtz et al., 2004). Collinear regions were identified using the nucmer module with 85% identity as the threshold with many-to-many alignment mode. Nucleotide substitutions in the collinear regions were analyzed using the dnadiff module. The collinear results were visualized using RIdeogram in the R package (Hao et al., 2020).

Comparative analysis of mitochondrial protein-coding genes and introns was also performed among the two *A. indica* individuals and seven other species of Orobanchaceae, including an autotrophic plant (*Lindenbergia philippensis*), three hemiparasitic plants (*Bartsia pedicularioides*, *Castilleja paramensis*, and *Schwalbea americana*), and three holoparasitic plants (*Orobanche crenata*, *O. gracilis* and *Phelipanche ramosa*), whose mitogenomes were characterized before (Fan et al., 2016).

### 3.4 Repeat identification and repeat-mediated recombination analysis

We used a Python script ROUSFinder2.0.py (Wynn and Christensen, 2019) to identify repeats (≥30 bp) in the *A. indica* mitogenome. Repeat units > 100 bp in size were used to assess repeat-mediated recombination. For all the repeats but the largest one (~16 kb), Illumina reads were mapped to sequences of the reference (the mitogenome we assembled) and potentially recombined conformations (repeat pair itself and flanking 300 bp single-copy sequences at both ends), and paired reads spanning these conformations were counted for calculating the recombination rate (the frequency of recombined conformations) for each repeat pair. The influence of plastid- and nuclear-derived reads was minimized by the same method in calculating the depth of coverage mentioned in section 3.1. We found one copy each of R3 and R5 (R3_b and R5_b) has an overlap of 3 bp, resulting in three predicted reference conformations and three predicted recombined conformations for each repeat pair (**Figure S2**), and we calculated the numbers of reads supporting each of these conformations and summarized in **Table 1**.

For the ~16 kb repeat pair, Illumina reads are too short to be used for assessing its recombination rate. We then designed the primers (**Table S3**) anchoring the regions flanking the repeat for the reference and potentially recombined conformations using Primer3 (Koressaar and Remm, 2007), performed long-fragment PCR amplification using KOD One™ PCR Master Mix (TOYOBO, Japan), and run agarose gel electrophoresis to verify the presence of repeat-mediated recombination.

### 3.5 Horizontal and intracellular gene transfer analysis for mitochondrial protein coding genes

For each of the 33 mitochondrial protein-coding genes of *A. indica*, we did phylogenetic analysis to infer its origin. Coding region sequences of as many as 18 other species, including species from the same family Orobanchaceae and its potential host family Poaceae, were downloaded from GenBank (https://www.ncbi.nlm.nih.gov/) (source details: **Supplementary Excel file 1**). Sequence alignment for each gene was performed with ClustalW (Thompson et al., 1997) and further adjusted manually. Phylogenetic trees were generated by RAxML v.8.2.11 using maximum likelihood (ML) method under the GTRGAMMAI model opting for one thousand bootstrap replicates. Either *Amborella trichopoda* or *Liriodendron tulipifera* was used as an outgroup in the phylogenetic analysis. Horizontal gene transfer (HGT) was inferred based on phylogenetic position of *A. indica*. HGT was identified if *A. indica* was grouped with non-Orobanchaceae species with high bootstrap support (>80%).

To detect the presence of intracellular gene transfer from the plastome to the mitogenome, the mitogenome of *A. indica* was searched against the *A. indica* plastome using BLASTN with the parameters of -evalue set to 1e-6. Due to massive gene loss in the *A. indica* plastome, the same BLASTN analysis was performed between the *A. indica* mitogenome and the plastome of the autotrophic plant *L. philippensi* (GenBank accession number NC_022859). Hits >100 bp in length and > 80% in identity were considered as potential intracellular gene transfer.

## Data availability statement

The datasets presented in this study can be found in online repositories. The names of the repository/repositories and accession number(s) can be found below: GenBank under the accession number TPA: BK061406.

## Author contributions

Conceptualization, RZ; methodology, YZ, RY, and JC; formal analysis, YZ and RY; data curation, YZ and JC; software, YZ; visualization, YZ; writing – original draft preparation, YZ and RZ; writing — review and editing, YZ, RY, and RZ; project administration, RZ and YL; funding acquisition, RZ. All authors have read and agreed to the published version of the manuscript.

## Funding

This work was supported financially by the National Natural Science Foundation of China (31811530297).

## Acknowledgments

We thank Choi Kyoung-Su and Park Seonjoo from Yeungnam University for sharing their mitogenome sequence of *Aeginetia indica*.

## Conflict of interest

The authors declare that the research was conducted in the absence of any commercial or financial relationships that could be construed as a potential conflict of interest.

## Publisher’s note

All claims expressed in this article are solely those of the authors and do not necessarily represent those of their affiliated organizations, or those of the publisher, the editors and the reviewers. Any product that may be evaluated in this article, or claim that may be made by its manufacturer, is not guaranteed or endorsed by the publisher.
