# Supplementary figures and images for: Highly active repeat-mediated recombination in the mitogenome of the holoparasitic plant Aeginetia indica
Source: Front Plant Sci. 2022 Sep 21;13:988368. doi: 10.3389/fpls.2022.988368 (PMC9532969; doi:10.3389/fpls.2022.988368)

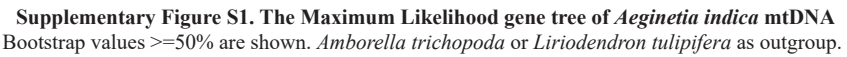

Supplement: Supplementary file 3 [file DataSheet_1.pdf]
