# Supplementary material for: Highly active repeat-mediated recombination in the mitogenome of the holoparasitic plant Aeginetia indica
Source: Front Plant Sci. 2022 Sep 21;13:988368. doi: 10.3389/fpls.2022.988368 (PMC9532969; doi:10.3389/fpls.2022.988368)
Supplement: Supplementary file 4 [file DataSheet_2.docx]

**Supplementary Information**

Highly active repeat-mediated recombination in the mitogenome of the holoparasitic plant *Aeginetia indica*

Yan Zhong^1^, Runxian Yu^2,3^, Jingfang Chen^1^, Ying Liu^1^, Renchao Zhou^1,*^

^1^ State Key Laboratory of Biocontrol and Guangdong Provincial Key Laboratory of Plant Resources, School of Life Sciences, Sun Yat-sen University, Guangzhou 510275, China

^2^ State Key Laboratory of Systematic and Evolutionary Botany, Institute of Botany, the Chinese Academy of Sciences, Beijing 100093, China

^3^ University of Chinese Academy of Sciences, Beijing 100049, China

*Author for correspondence. Email: [zhrench@mail.sysu.edu.cn](mailto:zhrench@mail.sysu.edu.cn)

**Supplementary Tables**

## Table S1. Summary of the mitochondrial gene content in Aeginetia indica

| Category | | Genes |
| --- | --- | --- |
| protein-coding genes | NADH dehydrogenase subunits | *nad1*, *nad2*, *nad3*, *nad4*, *nad4L*, *nad5*, *nad6*, *nad7*, *nad9* |
|  | cytochrome bc_1_ complex subunits | *cob* |
|  | cytochrome C oxidase subunits | *cox1*, *cox2*, *cox3* |
|  | ATP synthase subunits | *atp1*, *atp4*, *atp6*, *atp8*, *atp9* |
|  | cytochrome C maturation proteins | *ccmB*, *ccmC*, *ccmFC*, *ccmFN* |
|  | ribosomal protein large subunits | *rpl2*, *rpl5*, *rpl10*, *rpl16* |
|  | ribosomal protein small subunits | *rps3*, *rps4*, *rps12*, *rps13*, *rps14* |
|  | other protein-coding genes | *matR*, *mttB* |
| ribosomal RNAs | | *rrn5*, *rrn18*, *rrn26* |
| transfer RNAs | | *trnC-GCA*, *trnD-GUC-CP*, *trnE-UUC*, *trnF-GAA*, *trnG-GCC*, *trnH-GUG-CP*, *trnI-CAU* , *trnI-CAU-CP*, *trnK-UUU*, *trnM-CAU-CP* (3), *trnN-GUU-CP*, *trnP-UGG*, *trnQ-UUG*, *trnS-GCU*, *trnS-UGA*, *trnV-GAC-CP*, *trnW-CCA-CP*, *trnY-GUA* |
| pseudogenes | | ψ*rps10*, ψ*sdh4*, ψ*ndhB* * |

* intracellular gene transfer from the plastome

## Table S2. Protein-coding genes in the mitogenomes of *Aeginetia indica* and other seven species of Orobanchaceae.

| Genes | Ain  (this study) | Ain  (Choi and Park 2021) | Cpa | Bpe | Ocr | Ogr | Pra | Sam | Lph |
| --- | --- | --- | --- | --- | --- | --- | --- | --- | --- |
| # of genes | 33 | 34 | 34 | 34 | 32 | 32 | 34 | 34 | 35 |
| *atp1* | + | + | + | + | + | + | + | + | + |
| *atp4* | + | + | + | + | + | + | + | + | + |
| *atp6* | + | + | + | + | + | + | + | + | + |
| *atp8* | + | + | + | + | + | + | + | + | + |
| *atp9* | + | + | + | + | + | + | + | + | -^a^ |
| *ccmB* | + | + | + | + | + | + | + | + | + |
| *ccmC* | + | + | + | + | + | + | + | + | + |
| *ccmFc* | + | + | + | + | + | + | + | + | + |
| *ccmFn* | + | + | + | + | + | + | + | + | + |
| *cob* | + | + | + | + | + | + | + | + | + |
| *cox1* | + | + | + | + | + | + | + | + | + |
| *cox2* | + | + | + | + | + | + | + | + | + |
| *cox3* | + | + | + | + | + | + | + | + | + |
| *matR* | + | + | + | + | + | + | + | + | + |
| *mttB* | + | + | + | + | + | + | + | + | + |
| *nad1* | + | + | + | + | + | + | + | + | + |
| *nad2* | + | + | + | + | + | + | + | + | + |
| *nad3* | + | + | + | + | + | + | + | + | + |
| *nad4* | + | + | + | + | + | + | + | + | + |
| *nad4L* | + | + | + | + | + | + | + | + | + |
| *nad5* | + | + | + | + | + | + | + | + | + |
| *nad6* | + | + | + | + | + | + | + | + | + |
| *nad7* | + | + | + | + | + | + | + | + | + |
| *nad9* | + | + | + | + | + | + | + | + | + |
| *rpl2* | + | + | ψ | + | - | - | ψ | + | + |
| *rpl5* | + | + | + | + | + | + | + | - | + |
| *rpl10* | + | + | + | + | + | + | + | + | + |
| *rpl16* | + | + | + | + | + | + | + | + | + |
| *rps1* | - | - | - | - | ψ | - | ψ | - | - |
| *rps3* | + | + | + | + | + | + | + | + | + |
| *rps4* | + | + | + | + | + | + | + | + | + |
| *rps7* | - | - | - | ψ | - | + | - | ψ | - |
| *rps10* | ψ | ψ^a^ | + | + | ψ | ψ | + | + | + |
| *rps12* | + | + | + | + | + | + | + | + | + |
| *rps13* | + | + | + | + | + | ψ | + | + | + |
| *rps14* | + | + | + | + | + | + | + | + | + |
| *sdh3* | - | - | + | - | - | - | + | ψ | + |
| *sdh4* | ψ | ψ | ψ | ψ | ψ | ψ | - | + | + |

Species abbreviations: Ain: *Aeginetia indica*; Cpa: *Castilleja paramensis*; Bpe: *Bartsia pedicularioides*; Ocr: *Orobanche crenata*; Ogr: *Orobanche gracilis*; Pra: *Phelipanche ramose*; Sam: *Schwalbea americana*; Lph: *Lindenbergia philippensis*.

+: presence of a gene.

-: absence of a gene.

**ψ**: pseudogene.

^a^The absence of *atp9* in *Lindenbergia philippensis* may be the result of incomplete mitogenome assembly.

^b^*rps10* was originally annotated intact in Choi and Park 2021, but we found it was a pseudogene after careful inspection.

## Table S3. Primer sequences used to validate recombination mediated by the 16-kb repeat

| Primer | Sequences (5’ to 3’) | start | end |
| --- | --- | --- | --- |
| a_F | GACCTTCCTTCTACGCCTGTGGTTCTTG | 156520 | 156547 |
| a_R | ACATCTCGTTTGTTTGACTATTTCGTTTGC | 174254 | 174225 |
| b_F | AAGGGTTGGGCTGATAAAGACGGAATA | 368590 | 368616 |
| b_R | TGCGGAAGAAGCATAAGAGGTTAGGAA | 386179 | 386152 |

a_F and a_R are primers for repeat_1 (R1_a); b_F and b_R are primers for repeat_1 (R1_b). a_F and b_R, and b_F and a_R were primers used to amplify the repeats in the recombined conformations.

**Supplementary Figures**


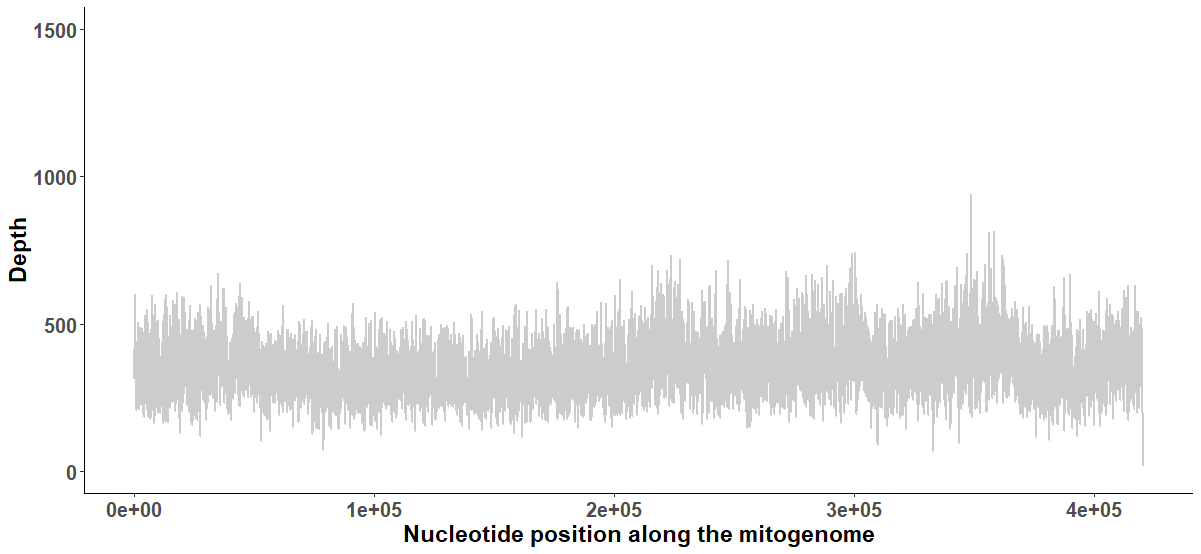


## Figure S1. The sequencing depth across the *Aeginetia indica* mitogenome.


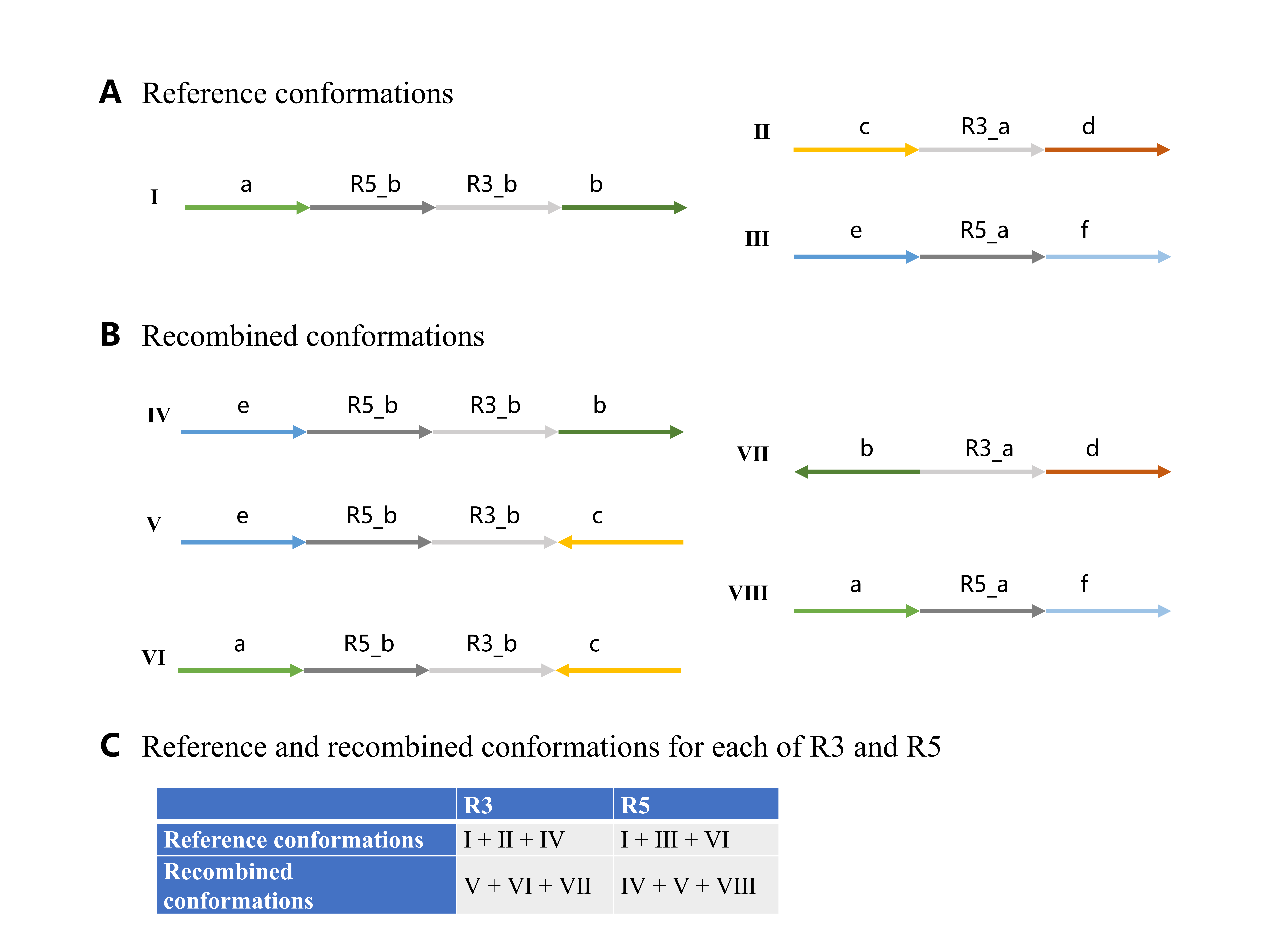


## Figure S2. The reference and predicted recombined conformations involving R3 and R5 repeat pairs in the *Aeginetia indica* mitogenome.

a-f represent the 300 bp sequences flanking R3 and R5 repeat pairs. Different colors represent unique flanking sequences and the arrows represent the sequence orientation.A. The reference conformations involving R3 and R5 repeat pairs; B. The recombined conformations involving R3 and R5 repeat pairs; C. The reference and recombined conformations used to calculate recombination frequency for R3 and R5 repeat pairs in Table 1.
